# Serum Amphiregulin and Heparin-Binding Epidermal Growth Factor as Biomarkers in Patients with Idiopathic Inflammatory Myopathy

**DOI:** 10.3390/jcm10163730

**Published:** 2021-08-22

**Authors:** Norio Hanata, Yasuo Nagafuchi, Yusuke Sugimori, Satomi Kobayashi, Yumi Tsuchida, Yukiko Iwasaki, Hirofumi Shoda, Keishi Fujio

**Affiliations:** 1 Department of Allergy and Rheumatology, Graduate School of Medicine, The University of Tokyo, Tokyo 113-8655, Japan; n_hanata@yahoo.co.jp (N.H.); nagafuchi@g.ecc.u-tokyo.ac.jp (Y.N.); ysugimori-tky@umin.ac.jp (Y.S.); satokobayashi.tky@gmail.com (S.K.); yumitsuchida@g.ecc.u-tokyo.ac.jp (Y.T.); yu_nyan@saitama-med.ac.jp (Y.I.); FUJIOK-INT@h.u-tokyo.ac.jp (K.F.); 2 Department of Functional Genomics and Immunological Diseases, Graduate School of Medicine, The University of Tokyo, Tokyo 113-8655, Japan

**Keywords:** idiopathic inflammatory myopathy, amphiregulin, heparin-binding epidermal growth factor, interstitial lung disease

## Abstract

Background. The epidermal growth factors amphiregulin (AREG) and heparin-binding epidermal growth factor (HB-EGF) are implicated in the pathogenesis of several autoimmune diseases, but their clinical and pathological roles in idiopathic inflammatory myopathy (IIM) are unclear. Methods. Serum AREG and HB-EGF levels were measured by ELISA in patients with IIM (*n* = 37), systemic sclerosis (*n* = 17), and rheumatoid arthritis (*n* = 10), and for seven age- and sex-matched healthy controls (HCs). Associations between serum AREG or HB-EGF levels and the clinical parameters were analyzed. Results. Serum AREG levels in IIM patients were significantly elevated compared to those in HCs (median, 20.7 and 10.7 pg/mL, respectively; *p* = 0.025). In particular, serum AREG levels in IIM patients with interstitial lung disease (ILD) were higher than those of HCs (22.4 pg/mL, *p* = 0.027). The disease duration in patients with elevated serum AREG levels was significantly shorter compared to those who had normal serum AREG levels (7 and 21 months, respectively; *p* = 0.0012). Serum HB-EGF levels were significantly increased in IIM patients with elevated CK levels (136.2 pg/mL; *p* = 0.020) and patients with anti-Mi-2 antibody (183.7 pg/mL; *p* = 0.045) compared to those in HCs (74.9 pg/mL). Conclusion. These results suggested that AREG could be a promising biomarker associated with early-phase IIM-related ILD, and that HB-EGF expression was associated with muscle injury and regeneration in IIM.

## 1. Introduction

The pathogenesis of idiopathic inflammatory myositis (IIM) is based on dysregulation of the immune system [1]. Besides myositis, systemic involvements are common in IIM and are closely associated with the prognosis. In particular, interstitial lung disease (ILD) in IIM can be resistant to immunosuppressive therapies and results in poor prognosis [2]. Several processes are associated with ILD progression in IIM. Recent analysis showed that bronchoalveolar lavage fluids of IIM patients with ILD were enriched in antigen-specific CD4^+^ T cells [3]. In addition, macrophage activation assessed using soluble CD163 as a biomarker was reported to be important in IIM-related ILD [4]. Macrophages not only contribute to autoantigen presentation, but also produce inflammatory mediators and pro-fibrotic mediators, such as transforming growth factor (TGF)-beta. However, the precise pathogenic mechanisms of IIM-related ILD remain unclear, and biomarkers capable of predicting disease activities and prognosis are required.

The epidermal growth factor (EGF) family includes structurally similar cell regulatory proteins that play important roles in cellular proliferation, differentiation, and apoptosis. In the context of idiopathic lung fibrosis (IPF), EGF receptor (EGFR)-related signaling drives fibrosis via activation of myofibroblasts. Amphiregulin (AREG) and heparin-binding epidermal growth factor (HB-EGF) are also members of the EGF family [5,6]. AREG is involved in a wide variety of physiological and pathological processes, including cell differentiation and proliferation, tissue repair, tumor development, and inflammation [5,7,8]. Furthermore, AREG plays an essential role in pathogenesis of TGF-beta-induced pulmonary fibrosis [9]. AREG binds to EGFR/ErbB2 heterodimers and induces transduction of a potent signal via tyrosine phosphorylation of EGFR [10]. Increased AREG levels in peripheral blood and tissues were reported for several autoimmune diseases, such as rheumatoid arthritis (RA) [11], systemic lupus erythematosus (SLE) [12], Sjogren’s syndrome [13], and psoriasis [14]. Similarly, HB-EGF plays critical roles in proliferation, migration, differentiation, tissue repair, and regeneration [5,15]. In the context of RA, HB-EGF-positive macrophages in synovial tissues showed pro-inflammatory phenotypes and could promote fibroblast invasiveness [16,17]. HB-EGF is also related to the pathogenesis of lupus nephritis [18,19]. Bollée et al. showed that HB-EGF expression was induced in podocytes of patients with proliferative glomerulonephritis along with increased phosphorylation of the EGFR/ErbB1 receptor [18]. HB-EGF is also implicated in the pathogenesis of systemic sclerosis (SSc) [20]. Together, these reports suggest that AREG and HB-EGF are related to the pathogenesis of autoimmune diseases and tissue fibrosis. Although both AREG and HB-EGF are involved in muscle repair processes in mice [21,22], little is known about their roles in IIM.

To examine the roles of AREG and HB-EGF in IIM pathogenesis and to assess their utility as novel biomarkers in IIM, in this study we measured serum AREG and HB-EGF levels in IIM patients and evaluated their association with clinical parameters.

## 2. Materials and Methods

### 2.1. Patients and Healthy Controls

Serum samples were obtained from patients with IIM (*n* = 37), SSc (*n* = 17), and RA (*n* = 10) who were admitted to the Department of Allergy and Rheumatology at the University of Tokyo Hospital between April 2017 and March 2019. IIM was diagnosed based on Bohan and Peter criteria for the diagnosis of PM and DM [23] and the European League Against Rheumatism/American College of Rheumatology (EULAR/ACR) classification criteria for adult and juvenile IIM [24]. SSc and RA were diagnosed according to the latest version of EULAR/ACR classification criteria [25,26]. None of the participants had active infection or malignancy. The demographic and clinical data retrospectively collected from patient records included age, sex, disease duration, whether or not the patients were under treatment or had ILD, vital capacity as percent of predicted values (%VC), and diffusing capacity for carbon monoxide as percent of predicted values (%DLCO), as well as serum levels of creatine kinase (CK), sialylated carbohydrate antigen Krebs von den Lungen-6 (KL-6), and autoantibodies. As healthy controls (HCs), serum samples from age- and sex-matched healthy donors collected from 2013 to 2015 were used (*n* = 7). All participants provided written informed consent prior to enrollment. This study was conducted in accordance with the latest version of the Declaration of Helsinki and was approved by the Ethical Committee of the University of Tokyo Hospital (number 11592 and G3582).

### 2.2. Measurement of Serum AREG and HB-EGF Levels

All blood samples were collected by venipuncture and clotted for 30 min at room temperature. Serum samples were then separated by centrifugation at 3000 rpm for 15 min and distributed in sterile tubes for storage at −30 °C until analysis. Serum AREG and HB-EGF levels were measured by ELISA (Human Amphiregulin (detection range; 15.6–1000 pg/mL) or HB-EGF Quantikine (detection range; 7.8–500 pg/mL) ELISA Kits from R&D Systems, Minneapolis, MN, USA, respectively) according to the manufacturer’s protocol. Each sample was tested in duplicate.

### 2.3. Statistical Analysis

GraphPad Prism9 (GraphPad Software, San Diego, CA, USA) was used for statistical analysis. An unpaired *t*-test was used for comparisons between two groups, and 1-way ANOVA with Tukey’s multiple comparison test was used for comparisons between three or more groups. Fisher’s exact test was used for categorical data analysis. Correlation was evaluated by Spearman’s correlation coefficient. *p* values < 0.05 were considered statistically significant.

## 3. Results

### 3.1. Baseline Characteristics

Demographic data and characteristics of patients and controls are listed in Table 1. No significant differences were observed in age or sex for each disease group compared to HCs.

### 3.2. Elevated Serum AREG Levels in Patients with IIM-Related ILD

**Figure 1:**
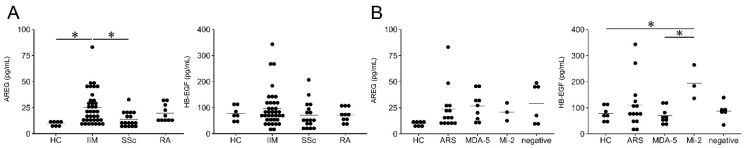
Serum levels of AREG and HB-EGF in HC and patients with IIM. (**A**) Serum levels of AREG (left) and HB-EGF (right) in HC and patients with IIM, SSc, and RA. (**B**) Serum levels of AREG (left) and HB-EGF (right) in HC, patients with anti-ARS, anti-MDA-5, and anti-Mi-2 antibodies, and patients who were antibody-negative. Groups with ≥3 patients were analyzed. HC: healthy controls; IIM: idiopathic inflammatory myopathy; SSc: systemic sclerosis; RA: rheumatoid arthritis; ARS: aminoacyl-tRNA synthetase; MDA5: melanoma differentiation-associated gene 5 protein; AREG: amphiregulin; HB-EGF: heparin-binding epidermal growth factor. Differences were analyzed by 1-way ANOVA followed by Tukey’s multiple comparison test: * *p* < 0.05.

Serum AREG levels were significantly higher for patients with IIM compared with those for HCs and patients with SSc (IIM: median, 20.7 pg/mL (IQR: 11.8–31.9); HC: median, 10.7 pg/mL (IQR: 8.3–11.5); *p* = 0.025; SSc: median, 11.8 pg/mL (IQR: 8.7–19.0); *p* = 0.023) (Figure 1A). With respect to autoantibodies, there was no significant difference in serum AREG levels among IIM patients and the types of myositis-specific antibodies (anti-ARS, anti-MDA-5, and anti-Mi-2 antibodies) (Figure 1B). 

**Figure 2:**
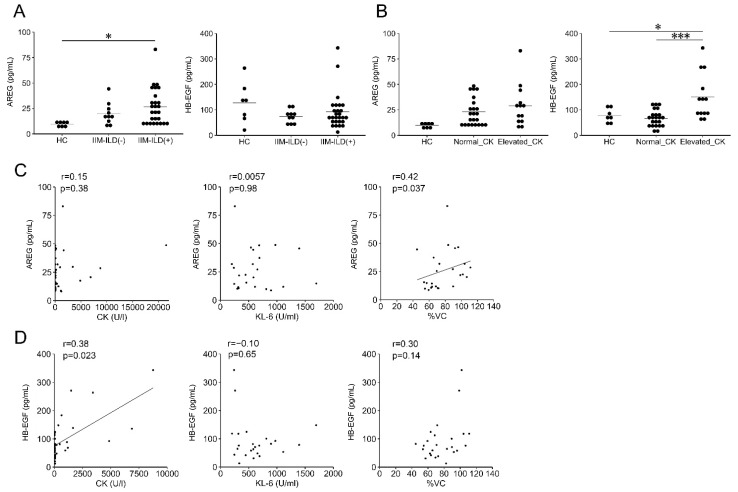
Serum levels of AREG and HB-EGF, and correlation with CK and KL-6 serum levels in patients with IIM. (**A**) Serum AREG (left) and HB-EGF (right) levels in HC and IIM patients with and without ILD. (**B**) Serum AREG (left) and HB-EGF (right) levels in HC, IIM patients with normal CK levels, and IIM patients with elevated CK levels. Elevated CK levels were defined as those exceeding the upper limits of the normal range of CK values (248 U/L and 153 U/L for males and females, respectively). (**C**) Correlation between CK levels and serum AREG in patients with IIM (left), correlation between KL-6/%VC levels and serum AREG in IIM patients with ILD (middle and right). (**D**) Correlation between CK levels and serum HB-EGF in patients with IIM (left), correlation between KL-6/%VC levels and serum HB-EGF in IIM patients with ILD (middle and right). * *p* <0.05, *** *p* <0.001.

Notably, serum AREG levels in IIM patients with ILD were significantly higher than those of HCs (IIM patients with ILD: median, 22.4 pg/mL (IQR: 11.8–34.6); HCs: median, 10.7 pg/mL (IQR: 8.3–11.5); *p* = 0.027) (Figure 2A). Meanwhile, no significant elevation in serum AREG levels was observed in IIM patients with or without serum CK elevation (HC: median, 10.7 pg/mL (IQR: 8.3–11.5); IIM patients with normal CK levels: median, 20.1 pg/mL (IQR: 11.7–29.5); IIM patients with elevated CK levels: median, 28.5 pg/mL (IQR: 14.8–31.9)) (Figure 2B). Serum AREG levels showed no significant correlation with other serum markers, including CK (*r* = 0.15, *p* = 0.38) or KL-6 (*r* = 0.0057, *p* = 0.98) (Figure 2C). Next, clinical parameters were compared between patients with and without elevated serum AREG levels (Table 2). Disease duration in patients with elevated serum AREG levels was significantly shorter compared to those with normal serum AREG levels (IIM patients with elevated serum AREG levels: median, 7 months (IQR: 1–18); those with normal serum AREG levels: median, 21 months (IQR: 8–93); *p* = 0.0012). In addition, %VC positively correlated with serum AREG levels (*r* = 0.42, *p* = 0.037) (Figure 2C). Taken together, serum AREG levels were elevated in IIM-related ILD patients, and in particular were associated with shorter disease duration and higher %VC. These results suggested that serum AREG levels could be a biomarker of early-phase IIM-related ILD.

### 3.3. Correlation of Serum HB-EGF Levels with Elevated CK

Next, serum HB-EGF levels were analyzed in IIM patients. Although no significant differences in serum HB-EGF levels were observed among the four groups (HCs, IIM, SSc, and RA) (Figure 1A), serum HB-EGF levels of IIM patients with anti-Mi-2 antibodies were significantly higher compared with those of HCs and IIM patients with anti-MDA-5 antibodies (IIM patients with anti–Mi-2 antibodies: median, 183.7 pg/mL (IQR: 159.9–223.8); HCs: median, 74.8 pg/mL (IQR: 56.8–98.0), *p* = 0.045; IIM patients with anti-MDA-5 antibody: median, 63.1 pg/mL (IQR: 48.2–81.3); *p* = 0.021) (Figure 1B). In contrast to AREG, serum HB-EGF levels were not substantially different between HC and IIM patients with and without ILD (HCs: median, 74.9 pg/mL (IQR: 56.8–98.0); IIM patients without ILD: median, 90.6 pg/mL (IQR: 71.7–138.1); IIM patients with ILD: median, 75.9 pg/mL (IQR: 50.8–106.5)) (Figure 2A). Notably, serum HB-EGF levels were significantly increased in IIM patients with elevated serum CK levels compared to those of HCs and IIM patients with normal CK levels (IIM patients with elevated CK levels: median, 136.2 pg/mL (IQR: 88.8–183.7); HCs: median, 74.9 pg/mL (IQR: 56.8–98.0); *p* = 0.020, IIM patients with normal CK levels: median, 64.5 pg/mL (IQR: 42.7–81.8); *p* < 0.001) (Figure 2B). Moreover, a significant positive correlation between serum CK levels and HB-EGF levels was observed (*r* = 0.38, *p* = 0.023) (Figure 2D). We then compared clinical parameters between IIM patients with and without elevation of serum HB-EGF levels (Table 2). No significant difference was observed between the two groups, except for the ratio of patients having elevated serum CK levels, as mentioned above. Taken together, serum HB-EGF levels were correlated with serum CK levels and could have potential as a biomarker of muscle injury and regeneration.

### 3.4. Relationship between AREG and HB-EGF

Finally, we analyzed the relationship between serum AREG and HB-EGF levels. No significant correlation between serum AREG and HB-EGF levels was observed in IIM patients (*r* = 0.10, *p* = 0.54). Furthermore, there was no difference in serum HB-EGF levels between IIM patients with and without serum AREG elevation and vice versa (Table 2). Therefore, serum AREG and HB-EGF levels could reflect different aspects of IIM pathophysiology, suggesting that a combination of these biomarkers could be a promising approach to understand the clinical characteristics of IIM.

## 4. Discussion

AREG and HB-EGF are members of the EGF family and share the same receptors, EGFR and ErbB2. Results of the present study suggested that the association with clinical parameters of IIM clearly differed between AREG and HB-EGF. Serum AREG levels were elevated in IIM-related ILD and were associated with shorter disease duration and higher %VC. These results suggested that serum AREG levels were elevated during the early phase of IIM-related ILD. Serum HB-EGF levels correlated with serum CK levels in IIM patients and could reflect the extent of muscle injury and regeneration. In IIM patients, muscle and extra-muscle inflammation are not always proportional. For example, IIM patients with anti-Mi2 antibody usually show higher CK elevation without ILD, whereas anti-MDA5 antibody-positive patients often develop clinically amyopathic dermatomyositis with rapidly progressing ILD [1,2]. We considered that serum AREG and HB-EGF levels are related to different inflammatory processes and locations in IIM and represent different aspects of IIM pathophysiology.

ILD is a problematic feature of IIM. IIM-related ILD is often refractory to treatment and is a risk for poor prognosis. The pathogenesis of IIM-related ILD is not fully understood. Recent studies that examined BALF of IIM-related ILD patients revealed pathogenic roles of T cell subpopulations [3]. Various sources of AREG production in the immune system were reported previously. In the context of airway fibrosis, memory Th2 cells produced AREG in response to IL-33 [27]. AREG is also produced by regulatory T cells, mast cells, and innate lymphoid cell 2, and are also involved in tissue repair. In the case of IIM-related ILD, subpopulations of T cells could produce AREG in response to epithelial damage signals. Furthermore, fibroblasts and dendritic cells are also reported to be cellular sources of AREG [9,28]. Zhou et al. reported that AREG expression was induced by TGF-beta stimulation and could regulate TGF-beta-induced fibroblast proliferation and pulmonary fibrosis through activation of the EGFR signaling pathway [9]. Ding et al. reported that AREG was specifically expressed in bone marrow-derived CD11c^+^ cells and promoted bleomycin-induced pulmonary fibrosis via fibroblast proliferation, survival, and myofibroblast differentiation [28]. Since ILD is likely caused by dysregulation of processes associated with tissue repair and fibrosis, AREG could contribute to progressive fibrosis in IIM-related ILD. In the present study, we found that serum AREG levels were upregulated during the early phase of IIM-related ILD. In general, serum levels of KL-6, a well-known marker of ILD, negatively correlates with %VC in patients with ILD. Interestingly, serum AREG levels did not correlate with serum KL-6 levels and positively correlated with %VC in IIM patients with ILD in our study. KL-6 is a high molecular weight (>1000 K) glycoprotein expressed on the surface membrane of type 2 alveolar pneumocytes, and an elevated level of serum KL-6 may result from the alveolar cell injury and destruction of lung vasculature [29,30]. In active ILD, KL-6 was within the normal range in approximately 30% of patients [31]. Thus, AREG can be detected in serum earlier than KL-6 in ILD because of its lower molecular weight, and AREG can be a promising biomarker associated with early-phase IIM-related ILD. As earlier detection and intervention of IIM-related ILD are associated with a better prognosis, serum AREG levels could be used as a good biomarker for predicting prognosis. In addition, based on the potentially pivotal role that dysregulation of AREG and EGFR signaling may play in pulmonary fibrosis pathogenesis, AREG could be a potential therapeutic target for IIM-related ILD. Further studies are warranted to identify the dominant cellular sources of AREG and its contribution to fibrosis in IIM-related ILD. Although anti-MDA5 antibody positive myositis is associated with rapidly progressive ILD [1,2], serum AREG levels of these patients was not increased significantly in multiple comparisons test. One possible reason might be the early detection and treatment initiation of anti-MDA5 positive IIM patients in our hospital. Further study is needed to increase the number of patients to characterize the difference of serum AREG levels among patients with different types of myositis-specific antibodies.

Progressive muscle weakness and atrophy are another problem in IIM. Aggressive muscle inflammation, necrosis, and regeneration processes result in muscle weakness and loss of ability to perform activities of daily living. Although immunosuppressive therapy, including steroids, can control muscle inflammation, restoration of muscle strength requires significant effort due to disuse and sometimes steroid-induced myopathy. Several immune cells, cytokines, autoantibodies, and complement factors play orchestrated roles in the pathogenesis of muscle inflammation and degeneration. HB-EGF is released from several types of cells, including monocytes and macrophages. In RA, HB-EGF-positive macrophages show pro-inflammatory phenotypes and promote fibroblast invasiveness [16]. In addition, HB-EGF is widely expressed throughout the body, including in human skeletal muscle [6], and in mice exercise upregulates HB-EGF expression in skeletal muscle [32]. In the present study, serum HB-EGF levels were significantly higher in IIM patients with elevated CK levels, which reflects injured muscle cells and infiltration of pro-inflammatory macrophages. Interestingly, HB-EGF promotes *Galgt*2 expression and expression of *Galgt*2-inducible genes in muscle to promote normal muscle development [22]. These findings suggested that HB-EGF could be involved in muscle regeneration processes in IIM.

This study has some limitations. First, the sample sizes were relatively small, and did not include several types of IIM, such as immune-mediated necrotizing myopathy. Second, tissue expression of AREG and HB-EGF in IIM patients, and histopathological finding of these patients were not examined, and the sources of these proteins and their correlation with histopathological findings were not clear. Although further studies will be required to address these points and define their clinical significance in IIM, the results of the present study do suggest potential roles for AREG and HB-EGF in IIM pathogenesis.

## 5. Conclusions

In conclusion, here we found that serum AREG levels were elevated in IIM patients, particularly those with ILD. Serum AREG levels were associated with shorter disease duration and higher %VC, suggesting that serum AREG could be used as a biomarker of early phase IIM-related ILD. Meanwhile, serum HB-EGF levels were increased in IIM patients with elevated CK levels, suggesting that HB-EGF could indicate muscle injury and regeneration in IIM. Together, the results indicated that AREG and HB-EGF play different roles in the pathogenesis of IIM and provide insights into the mechanisms underlying clinical varieties of IIM.

## Figures and Tables

**Table 1:** Demographic data and baseline characteristics of patients and controls.

	IIM (*n* = 37)	*p*-Value	SSc (*n* = 17)	*p*-Value	RA (*n* = 10)	*p*-Value	HC (*n* = 7)
Female, *n* (%)	26 (71.3)	0.41	16 (94.1)	0.52	8 (80)	0.78	6 (85.7)
Age, years (range)	59.0 (51.0–71.0)	0.75	70.0 (55.0–75.0)	0.13	69.0 (55.5–76.8)	0.16	57 (56.5–60.0)
Disease duration, months (range)	16 (7–84)		60 (34–288)		53 (14–177)		-
Untreated, *n* (%)	14 (37.8)		6 (35.3)		0 (0)		-
ILD, *n* (%)	27 (72.9)		10 (58.8)		3 (30)		-
CK, mean (S.D.), U/L	1520 (3955)		n.d.		n.d.		n.d.
KL-6, U/mL (range)	506 (289–687)		533 (371–1144)		271 (212–372)		n.d.
%VC, % (range)	79.6 (65.1–96.8)		76.7 (67.0–84.5)		100.9 (77.5–132.6)		n.d.
%DLCO, % (range)	74.7 (64.3–90.8)		80.4 (71.0–93.1)		82.4 (72.6–92.2)		n.d.
	IIM (*n* = 37)	N (%)	SSc (*n* = 17)	N (%)	RA (*n* = 10)	N (%)	
Autoantibody profile, *n* (%)	ARS	14 (37.8)	Scl-70	6 (35.3)	RF	9 (90)	
	Jo-1	5 (13.5)	Centromere	9 (52.9)	ACPA	9 (90)	
	PL-7	3 (8.1)	RNA polymerase III	2 (11.8)			
	PL-12	1 (2.7)					
	EJ	2 (5.4)					
	Unknown	3 (8.1)					
	MDA-5	9 (24.3)					
	TIF-1γ	1 (2.7)					
	Mi-2	3 (8.1)					
	SRP	1 (2.7)					
	HMG-CR	1 (2.7)					
	M2	2 (5.4)					
	Negative	6 (16.2)					

Data are reported as median (IQR) unless stated otherwise. n.d.: no data. *p* values were calculated using Fisher’s exact test to assess the proportion of females, and unpaired t tests were used to assess differences in age between healthy controls and patients in each disease group. Disease duration was defined as the duration from the diagnosis of IIM to the serum sample collection. IIM: idiopathic inflammatory myopathy; SSc: systemic sclerosis; RA: rheumatoid arthritis; HC: healthy controls; ILD: interstitial lung disease; CK: creatine kinase; KL-6: Sialylated carbohydrate antigen Krebs von den Lungen-6; VC: vital capacity; DLCO: carbon monoxide diffusing capacity; ARS; aminoacyl-tRNA synthetases; MDA5: melanoma differentiation-associated gene 5 protein; TIF1-ɤ: transcriptional intermediary factor 1ɤ; SRP: signal recognition particle; HMGCR: 3-hydroxy-3-methyl coenzyme A reductase protein; M2: mitochondrial M2; RF: rheumatoid factor; ACPA: anti-cyclic citrullinated peptide antibody; IQR: interquartile range.

**Table 2:** Clinical and laboratory parameters in IIM patients with or without elevated serum AREG/HB-EGF levels.

	AREG			HB-EGF		
	Normal (*n* = 30)	Elevated (*n* = 7)	*p* Value	Normal (*n* = 31)	Elevated (*n* = 6)	*p* Value
Female, *n* (%)	21 (70.0)	5 (71.4)	1.00	22 (71.0)	4 (66.7)	1.00
Age, years (range)	58 (50–68)	71 (63–77)	0.064	59 (51–71)	61 (57–68)	0.76
Disease duration, month (range)	21 (8–93)	7 (1–18)	0.001	16 (7–84)	18 (9–41)	0.79
Untreated, *n* (%)	11 (36.7)	3 (42.9)	1.00	11 (35.5)	3 (50)	0.65
ILD, *n* (%)	21 (70.0)	5 (85.7)	0.08	7 (22.6)	3 (50)	0.31
CK, U/L	69 (46–639)	186 (42–1571)	0.43	65 (73–192)	2451 (850–6009)	0.16
Elevated CK, *n* (%)	10 (34.5)	3 (42.9)	0.69	7 (23.3)	6 (100)	<0.001
KL-6, U/mL (range)	453 (259–644)	634 (550–900)	0.27	557 (321–687)	248 (231–617)	0.93
Elevated KL-6, *n* (%)	9 (40.9)	5 (83.3)	0.16	13 (54.2)	1 (25.0)	0.60
%VC, % (range)	83 (68–102)	71 (57–87)	0.23	77 (64–90)	100 (92–105)	0.14
%DLCO, % (range)	76 (65–94)	70 (57–78)	0.24	74 (64–85)	94 (85–99)	0.16
AREG, pg/mL (range)	16.8 (11.6–25.2)	46.5 (45.1–48.6)	<0.001	20.1 (11.7–31.9)	24.6 (16.3–29.4)	0.50
HB-EGF, pg/mL (range)	78.7 (49.5–116.6)	78.4 (69.5–115.6)	0.59	70.9 (50.8–90.6)	223 (157–269)	0.0055

Data are reported as median (IQR) unless stated otherwise. Numbers may not add up to the expected total since data were not available for some variables. The upper limits of normal range of CK: 248 U/L (male), 153 U/L (female); KL-6: 500 U/mL. Elevated levels of serum AREG was defined as ≥40 pg/mL based on the finding that the serum AREG levels of any patient with disease controls (SSc and RA) did not exceed 40 pg/mL. Elevated levels of serum HB-EGF (≥133.2 pg/mL) was defined as greater than those of the mean for HC plus 2SD. Disease duration was defined as the duration from the diagnosis of IIM to the serum sample collection. IIM: idiopathic inflammatory myopathy; SSc: systemic sclerosis; RA: rheumatoid arthritis; ILD: interstitial lung disease; CK: creatine kinase; KL-6: Sialylated carbohydrate antigen Krebs von den Lungen-6; VC: vital capacity; DLCO: carbon monoxide diffusing capacity; AREG: amphiregulin; HB-EGF: heparin-binding epidermal growth factor. Each clinical parameter in the normal group and the elevated group of AREG/HB-EGF was analyzed using unpaired *t*-test for continuous variables and Fisher’s exact test for categorical variables.

## Data Availability

The data that support the findings of this study are available from the corresponding author upon reasonable request.
